# Effectiveness of counseling for anxiety and depression in mothers of children ages 0-30 months by community workers in Karachi, Pakistan: a quasi experimental study

**DOI:** 10.1186/1471-244X-10-57

**Published:** 2010-07-19

**Authors:** Niloufer S Ali, Badar S Ali, Iqbal S Azam, Ali K Khuwaja

**Affiliations:** 1 Department of Family Medicine, Aga Khan University Stadium Road, P. O. Box 3500, Karachi 74800, Pakistan; 2 Department of Family Medicine, Aga Khan University, Karachi, Pakistan, Stadium Road, P. O. Box 3500, Karachi 74800, Pakistan; 3 Department of Community Health Sciences, Aga Khan University, Karachi, Pakistan, Stadium Road, P. O. Box 3500, Karachi 74800, Pakistan; 4 Department of Family Medicine & Community Health Sciences, Aga Khan University Stadium Road, P. O. Box 3500, Karachi 74800, Pakistan

## Abstract

**Background:**

The prevalence of anxiety/depression is quite high during the perinatal period but unfortunately its detection and treatment have been less than satisfactory. Moreover, many women are reluctant to take pharmacotherapy for fear of excretion of drugs into their breast milk. This study assesses the effectiveness of counseling from minimally trained community health workers in reducing anxiety/depression, the rate of recurrence and the interval preceding recurrence in women during first two and a half years after childbirth.

**Methods:**

In a quasi-experimental study, community women from two under-privileged communities were trained in data gathering, teaching healthy child-rearing practices, basic counseling skills, and screening for anxiety/depression by using an indigenously developed questionnaire, the Aga Khan University Anxiety and Depression Scale (AKUADS). The diagnosis was further confirmed by a clinical psychologist using DSM IV criteria. After obtaining consent, 420 women were screened and 102 were identified as having anxiety/depression. Screening was carried out after 1, 2, 6, 12, 18, 24 and 30 months of a live birth. Only 62 out of 102 agreed to be counseled and received eight weekly sessions. AKUADS was re-administered at 4 weeks and 8 weeks after the beginning of counseling; this was followed by the clinical psychologist's interview for confirmation of response. After recovery, screening was continued every 3 months for detection of recurrence throughout the study period. Out of the women who had declined counseling 12 agreed to retake AKUADS after 4 and 8 weeks of diagnosis. Independent samples t-test, chi-square test, Repeated Measures ANOVA and Kaplan Meier technique were used for the analysis.

**Results:**

A significant decline in level of anxiety/depression was found in both the counseled and the non-counseled groups at 4 and 8 weeks (p-value < 0.001) but the counseled group fared better than the non-counseled for recovery, reduction in the rate of recurrence and increase in the duration before relapse.

**Conclusions:**

As our results cannot be generalized; further studies need to be carried out, to assess the benefit of incorporating minimal counseling skills in the training of community health workers.

## Background

Globally the prevalence of mental health and psychosocial problems is high during pregnancy and after birth. It is reported that almost one in four women in developing countries suffers from anxiety/depression around the period of childbirth [1], which can lead to a chronic or recurring depressive course throughout life [2]. A literature review of 143 research studies undertaken in 40 countries around the world reported that the prevalence of postpartum depression (PPD) in Asian countries ranged from 11% to 60.8% [3].

The mean overall prevalence of anxiety and depressive disorders in the Pakistani population has been reported to be 34% with higher rates in women than in men [4]. Ali et al have reported a prevalence of 30% among women of reproductive age group in a semi-urban community of Karachi, Pakistan [5]. Studies done in urban tertiary care settings in Pakistan, have reported figures ranging from 24% - 42% [6,7]. Other community-based studies from rural Pakistan have reported prevalence ranging from 28% - 36% in postpartum women [8,9]. A cohort study from rural Pakistan has reported persistent postpartum depression in 56% of those who developed PPD [10].

Depression after childbirth affects the health of the mother as well as the health, growth and development of the child [11]. Studies have revealed that perinatal depression is associated with poor growth, high risk of diarrhoea and reduced uptake of immunization [12,13].

Currently the detection and treatment of depression after childbirth is less than satisfactory and many women are reluctant to take pharmacotherapy for fear of excretion of drugs into their breast milk [14]. Studies from high income countries have reported that psychotherapeutic approaches, such as cognitive behaviour therapy, interpersonal therapy or problem solving therapy are effective treatments for depression [15]. A few randomized trials from low income and middle income countries have also reported similar findings [16,17]. Substantial decrease in depressive symptoms in women after miscarriage has been reported after six sessions of interpersonal counseling [18]. A randomized controlled study conducted by Rahman et al in a poor rural community in Pakistan has shown that integration of cognitive behaviour therapy (CBT) into routine work of community health workers more than halved the rate of depression in prenatally depressed women compared with those receiving routine care only. In addition to symptomatic relief, the intervention group had less disability, better overall social functioning and the benefit persisted after one year [19]. Bhar et al have reported that patients treated with either pharmacotherapy or cognitive therapy showed similar results [20].

No published findings to date suggest that antidepressant medication reduces future risk of depressive episodes after discontinuation [21], whereas cognitive therapy has been shown to provide protection against relapse and possible recurrence [22]. Hollon et al have reported higher percentages of relapses and recurrences among patients who were on pharmacotherapy than on cognitive therapy [23].

In a randomized controlled trial Ali et al. found significant improvement in women of reproductive age who were suffering from anxiety and depression after eight weekly counseling sessions by minimally trained community women [24]. Based on this experience, this study was conducted to assess the benefits of counseling from minimally trained community health workers working with anxious and depressed women during the first two and a half years after childbirth, and to assess the rate of recurrence and the interval preceding recurrence of anxiety/depression after counseling.

## Methods

### Study design, site and duration

This is a quasi-experimental study from an action research program to assess "The effect of postpartum anxiety and depression on early childhood growth and development," implemented in two semi-urban underprivileged communities (Qayoomabad and Manzoor Colony) of Karachi, a mega city of Pakistan from February 2004 to January 2007.

### Study participants and sample

A total of 420 consenting women (155 from Manzoor Colony and 265 from Qayoomabad) were enrolled after live childbirths. Out of these, 102 women (34 from Manzoor Colony and 68 from Qayoomabad) developed anxiety/depression during the study period. Detailed information about the recruitment process is given elsewhere [25].

### Instruments used

#### a. Aga Khan University Anxiety and Depression Scale (AKUADS)

This is a screening instrument for anxiety and depression developed from symptoms of anxious and depressed patients noted verbatim in the local lingua franca Urdu and validated in the community keeping the psychiatrist's interview as the gold standard [26,27]. It is a 25 item scale (13 psychological and 12 somatic items) that covers most of the clinical features considered characteristic of anxiety and depressive disorders. Each item has four response options (never, sometimes, often, always) scored from 0 to 3. At a cut-off score of 19 it has a sensitivity of 74%, specificity of 81%, a positive predictive value of 63% and a negative predictive value of 88% [27]. It has been used in several studies in Pakistan due to its near 100% linguistic validity [5,24-29]. As AKUADS is a screening instrument, diagnostic confirmation was obtained by a clinical psychologist's interview. Mothers who tested positive on AKUADS (score of 19 or above) and those who were marginally below the cut-off score i.e. with scores of 17 and 18 were interviewed by a clinical psychologist for confirmation of diagnosis according to DSM IV criteria. However, women with a score of 16 and below were considered as not anxious/depressed and were not interviewed by the clinical psychologist.

#### b. Socio-demographic questionnaire

This included mother's age, religion, ethnicity, education and occupation, husband's education and occupation, monthly household income (in Pakistani Rupees), ownership of house, total number of rooms, total number of household members, number of pregnancies, number of live births, number of abortions/stillbirths, number of deceased children, reasons for their deaths, whether the current pregnancy was planned or otherwise, if the subject had ever used or had intended to use contraception and had the autonomy to do so.

#### c. Home environment/Family relationship questionnaire

included presence or absence of a stressful home environment, satisfaction with current life, available family/social support, how household decisions were made, if the mother or the children were ever abused physically or verbally by any family member.

#### d. Post-natal questionnaire

recorded the gender, date, weight (in kg.), place of birth, qualification of the birth attendant, complications, if any, during or after birth in the mother or the newborn.

#### e. Early childhood development (ECD) tool

which comprised five major components of development i.e. gross motor, fine motor, language, cognitive and socio-emotional development.

### Selection and training of field workers as counselors

Women aged 18 years and above residing at the study sites, able to read and write Urdu (the lingua franca in Pakistan) and willing to be trained were identified. They were trained in administration of the screening instrument, The Aga Khan University Anxiety and Depression Scale (AKUADS), and the other study questionnaires mentioned above. They were also trained to provide counseling to mothers who were found to be anxious and depressed. The training extended over 5 half days per week, for four weeks, each session being of three hours. The trainers included two family practitioners, a psychiatrist and a clinical psychologist. The training encompassed basic information regarding anxiety/depression, stress/anger management and communication/counseling skills. Communication covered principles of active listening, probing and feedback, whereas counseling dealt with supportive, problem-solving and basic cognitive-behavioral techniques. The model used was participatory and facilitatory. Out of the 19 trained women, 11 were selected as counselors based on their ability to maintain confidentiality, communicate empathically and to gain permission from family to move freely in the community.

In addition, they were trained in monitoring the growth and development of the indexed baby by measuring weight, height and head circumference. They were also trained in using the early childhood development (ECD) tool and in providing information regarding healthy child-rearing practices which included: care of the cord, avoiding traditional harmful practices like application of surma (an eye cosmetic believed to heal eye infection and available as a fine powder of lead sulfide), promoting breast feeding, and advice on timely weaning and immunization.

### Enrolment and data collection

A field office was established at Qayoomabad and the counselors visited each house in Qayoomabad and adjacent sectors of Manzoor colony, and inquired about the date of the last menstrual period from women in the reproductive age group. Those found to be pregnant were informed of the objectives of the study and were invited to participate in the study after childbirth and written consent was obtained. This process of identification of pregnant women was carried on for the first two years of the study. The expected date of delivery was calculated from the date of the last menstrual period and the counselors started weekly home visits when the participant woman had reached the 36^th ^week of pregnancy, and this continued until childbirth. Consent was again obtained from mothers of live births before enrolling them in the study. The socio-demographic, home environment, family relationship and newborn postnatal questionnaires were administered within seven to ten days of childbirth by the counselors. Routine follow-ups were scheduled after 1, 2, 6, 12, 18, 24 and 30 months of childbirth for screening of anxiety/depression among mothers and monitoring of child growth/development. Verbal consent was obtained each time before administering AKUADS. Women with AKUADS scores of 17 or above were interviewed by the clinical psychologist for confirmation of anxiety/depression according to DSM IV criteria.

A total of 102 women were found to be anxious and depressed at least once during the study period. Among them 84 had only one episode, 16 had two episodes and 2 women suffered three episodes giving a total of 122 episodes. (Table 1).

**Table 1:** Distribution of episodes of anxious and depressed women with their AKUADS scores at the time of identification by month of identification and area of residence (n = 122)

AKUADS scores at the time of identification*	Month of Identification							All
		
	1	2	6	12	18	24	30	
**Manzoor Colony (MC)**								

Number of women Interviewed	155	147	112	94	76	56	17	155

19+	3	5	11	9	4	3	0	35

>16 & <19	0	0	0	1	1	1	1	4

**MC**	**3**	**5**	**11**	**10**	**5**	**4**	**1**	**39**

**Qayoomabad (QA)**								

Number of women Interviewed	265	255	223	173	150	109	59	265

19+	10	9	13	25	12	7	0	76

>16 & <19	1	0	3	0	1	1	1	7

**QA**	**11**	**9**	**16**	**25**	**13**	**8**	**1**	**83**

**All**	**14**	**14**	**27**	**35**	**18**	**12**	**2**	**122**

* Ten women with scores greater than 19 but clinically negative were counseled but have been excluded

**Figure 1:**
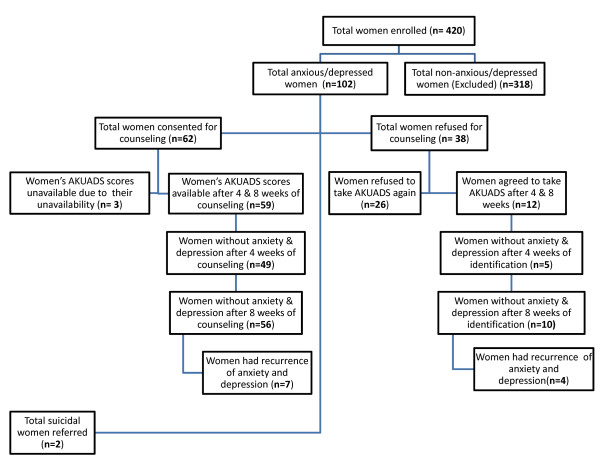
**Flow Chart of women identified as anxious and depressed**.

The number of episodes of anxiety/depression at 1 month, 2 months, 6 months, 12 months, 18 months, 24 months and 30 months were 14, 14, 27, 35, 18, 12 and 2 respectively. All women found to be anxious/depressed by the clinical psychologist or who scored 19 or above on AKUADS were offered weekly one-hour counseling sessions for eight weeks. Only 62 accepted; main reasons for refusal were objection from their husbands and in-laws and the social stigma attached to the diagnosis of a mental illness. Very basic cognitive behavioral therapy, supportive and problem-solving counseling was provided. Sessions were conducted at the client's residence on the day and time of her convenience. The counselors kept notes of their sessions and discussed these with the clinical psychologist on a daily basis initially, and once weekly as they became better trained and more confident. They also had easy access to the other members of the training team throughout the study period. All identified women whether they had agreed to counseling or not were requested to take the AKUADS at 4 and 8 weeks after being diagnosed. Those women whose AKUADS scores after 8 weeks of counseling were 16 and below were considered to have recovered and those with a score of 17 or above were interviewed by the clinical psychologist for the confirmation of persistence of anxiety/depression. Only five of them (n = 3 counseled; n = 2 non-counseled) did not recover after eight weeks of identification and were advised to seek pharmacological treatment. Those who had recovered from anxiety and depression were then followed regularly according to the study protocol for recurrence during the study period. Recurrence was considered when, at any follow-up visit the AKUADS score was found to be 17 and above, and confirmation was also obtained from the psychologist. Two suicidal patients were referred for treatment and were not included in the study. The flow of participants is shown in Figure 1.

All women enrolled in the program, irrespective of whether they were anxious/depressed or not or whether they were being counseled or not, were instructed in healthy child rearing practices and the growth and development of their indexed child was monitored.

### Data management and analysis

Data was double entered using EpiData (version 3.02) package; 10% of the records were randomly checked to assess the quality of data entry. The final data were analyzed using the statistical software package SPSS (Statistical Package for Social Sciences; version 15.0).

Independent samples t-test for the mean differences by counseling status (counseled and not counseled that had accepted to take AKUADS) were observed for quantitative variables like age, gravidity, baseline AKUADS scores, total number of rooms in the house, and total number of persons per household.

A chi-square test was used to observe the association between different socio-demographic categorical variables by counseling status. The categorical variables studied were the couple's education, mother tongue, history of domestic violence, religion, migrant status, ownership of house, past history of still births, gender of the newborn, qualification of the birth attendant, place of delivery and difficulty in breast feeding. Among the non-counseled group, same statistical techniques were used to assess the association of these variables with the groups who had refused counseling but had accepted taking AKUADS at 4 and 8 weeks and those who had refused to do so.

Repeated Measures ANOVA was used to see the difference in the AKUADS scores and an error chart was made with 95% confidence interval for mean AKUADS score at the time of initial diagnosis, and after 4 weeks and 8 weeks of diagnosis by counseling status. Pairwise comparisons were made using Bonferroni's method. Partial Eta square was used to report effect sizes for each component. The Kaplan Meier method was used to calculate the mean recurrence time with 95% confidence intervals for the counseled and the not counseled and log rank test was used to assess the significant difference in the mean recurrence time. A p-value of 0.05 was considered as significant.

## Results

Out of the 420 women enrolled, 102 women were found to be anxious and or depressed with a total of 122 episodes, based on the AKUADS score and supplemented by the clinical psychologist's interview. AKUADS scores after 4 and 8 weeks were only available for seventy one women i.e. 59 from the counseled and 12 from the not counseled group (Figure 1).

The association of different characteristics by counseling status was observed. No significant differences were found for variables such as age, level of education, past history of anxiety and depression, husband's education, religion, mother tongue, migrant status, ownership of house, total number of rooms, total number of persons per household, gravidity, past history of still birth, satisfaction with current life, domestic violence, gender of the newborn, qualification of the birth attendant and place of birth. The characteristics having a significant association were difficulty in breast feeding (χ^2^(2) = 11.824, p-value = 0.002) and study area (Fisher Exact(1), p-value = 0.049). The average AKUADS score at the time of diagnosis was significantly higher in those who had refused counseling as compared to those who had agreed to and received counseling (Z = -3.764, p-value < 0.001) (Table 2).

**Table 2:** Comparison of different characteristics of study participants by counseling status

*Characteristics*	*All* *(n = 71)*	*Counseled* *(n = 59)*	*Not-counseled* *(n = 12)*	*P value*
**Study Area**				0.049
Qayoomabad	47	36	11	
Manzoor Colony	24	23	1	

**Age Group**				0.077
				
<25	20	17	3	
25-29	27	24	3	
30-34	17	14	3	
35 & above	7	4	3	
				
Mean age (SD)	27.4 (5.0)	26.9 (4.7)	29.8 (5.9)	

**Educational Level**				0.075
				
Illiterate	18	15	3	
Can read & write	6	3	3	
Below matriculation	24	23	1	
Matriculation	12	10	2	
Intermediate & above	11	8	3	

**Past History of Anxiety or Depression**				0.207
				
Yes	9	9	0	
No	62	50	12	

**Difficulty in breast feeding**				0.002
No	57	51	6	
Yes				
Mother	9	7	2	
Baby	5	1	4	

AKUADS Scores when diagnosed as anxious and depressed*				<0.001
Mean (SD; Min. Score, Max. Score)	20.5 (6.4; 7, 57)	19.3 (3.6; 7, 27)	26.1 (11.9; 12, 57)	

* Based on the number of episodes all = 73, counseled = 60 and not counseled = 13

**Figure 2:**
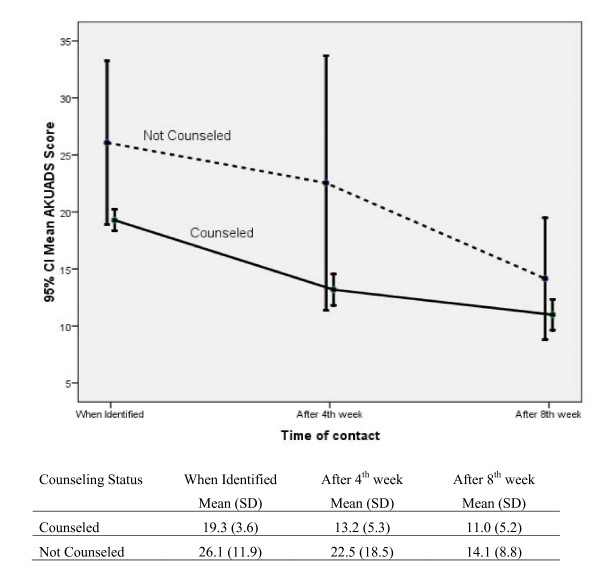
**Mean AKUADS Score (95% confidence interval) by Time of Contact in counseled and not counseled women**. (n = 71).

Repeated measures ANOVA of AKUADS scores of anxious/depressed women after the 4^th ^and 8^th ^weeks of identification showed significant effects of time (Time: F(2,142) = 37.89, p-value < 0.001; Partial Eta square = 0.348), interaction between time & counseling status (TimexCounseling Status: F(2,142) = 3.577, p-value = 0.031; Partial Eta square = 0.048) and counseling status (Counseling Status: F(1,71) = 14.071, p-value < 0.001; Partial Eta square = 0.165) (Figure 2).

Pair-wise comparisons of mean AKUADS scores using Bonferroni's method were made for all time points (when identified, after 4^th ^and 8^th ^weeks of identification) and were found significant (p-value < 0.001) for all time points pairs. Similarly, pair-wise comparisons for counseling status at each time point were made and mean AKUADS scores at the time of identification (p-value < 0.001) and after 4^th ^week of identification (p-value < 0.001) were found to be significant.

During the overall study period from February 1, 2004 to January 31, 2007, post-test data were available for 71 women diagnosed as anxious and depressed at induction in the study (n = 59 counseled; n = 12 non-counseled). Out of them 54 women had recovered after 4 weeks (n = 49 counseled; n = 5 non-counseled) and 66 women after 8 weeks. (n = 56 counseled; n = 10 non-counseled) (Figure 1).

**Figure 3:**
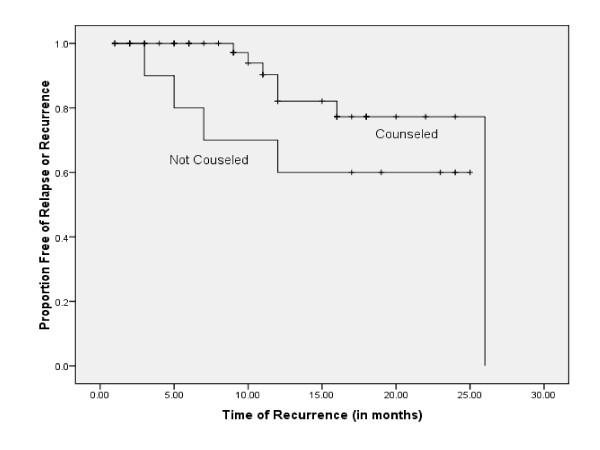
**Kaplan Meier Survival Curves estimating time until recurrence by Counseling Status (n = 11)**.

During the regular follow-up, recurrence of anxiety and depression was observed in 7 women among the counseled group and in 4 women among the non-counseled group. The earliest recurrence time in the counseled group was 9 months and the latest was 26 months as compared to the not counseled in which the earliest recurrence occurred at 3 months and the latest at 12 months. No significant difference was observed in the mean recurrence time between the counseled (Mean = 22.8 months, SE = 1.2 months; 95% C.I.: 20.4, 25.3) and not counseled group (Mean = 17.7 months, SE = 2.9; 95% C.I.: 12.0, 23.4) (Log Rank χ^2^(1) = 2.33, p-value = 0.127), (Figure 3).

## Discussion and Conclusions

The findings of this study suggest that after 4 and 8 weeks the scores of AKUADS, (both the counseled and not counseled groups) showed a significant decline from the initial scores, but the counseled group fared better than the not counseled. Rahman et al have reported that integration of a cognitive-behavior-therapy-based intervention by community health workers had substantially reduced the rate of depression in prenatally depressed women compared with those receiving routine care [19]. DeRubeis et al have also reported that cognitive therapy can be as effective as medications in the initial treatment of moderate to severe major depression [30].

Lack of autonomy is one of the known vulnerability factors for anxiety/depression and our results support this finding as the AKUADS scores at the time of identification were higher among the group that could not be counseled because of lack of permission from the family.

The significant decline in the not counseled group suggests several possibilities such as the fact that lapse of time since delivery itself tends to reduce anxiety/depression in women, or that it reflects the natural history of anxiety/depression that waxes and wanes. It could also be the result of enhanced social support because of regular visits from counselors, monitoring of growth and development of the indexed child and learning healthy child-rearing practices by all mothers. The later possibility also makes it difficult to ascertain the decline in scores of the counseled group as resulting from purely counseling to the actual or perceived social support experienced by the group.

A meta-analysis published in The Cochrane Database of Systemic Reviews 2007 suggests that psychosocial and psychological interventions are both effective treatment options for postpartum depression [31]; our results also suggest that probably psycho-social support alone and in combination with counseling could benefit postpartum women with anxiety and depression, the separate contribution of each one cannot be determined as the data is from an action research program and not a randomized controlled trial.

More recently, a randomized controlled study has suggested that interpersonal psychotherapy ameliorates depression during pregnancy and prevents depressive relapse and improves social functioning up to six months postpartum [32]. Varying recurrence rates have been reported [33,34] for various types of psychotherapy, from 26%-67%. It has been reported that cognitive behavior treatment (CBT) resulted in a significantly lower relapse rate (40%) at a 6 year follow up [35] than pharmacological management (90%). In our study, which was conducted over a period of 30 months, we found an overall recurrence rate of 16.4%, with a marginally significant difference (p-value = 0.051) between the counseled (12.3%) and the non counseled groups (28.6%). The recurrence rate in the counseled group is lower than that mentioned in the studies referred above, as probably our study population had mild to moderate depression due to the fact that suicidal patients and those who had not responded to eight sessions of counseling were referred for treatment; for ethical reasons these patients had continued to be counseled but were not included in the analysis.

In our study we also observed that minimum time interval before recurrence was longer in the counseled i.e. 9 months as compared to the not counseled in which it was 3 months. Cognitive therapy has demonstrated an enduring effect that prevents the return of symptoms after successful treatment [36]. Dobson et al have also reported that cognitive therapy has an enduring effect and is a less expensive and longer-lasting alternative to medications [37]. Ali et al have consistently found an encouraging response to counseling by minimally trained community women in underprivileged communities [24,38].

One of the limitations of our study was that the Ethics Committee of the Aga Khan University declined to approve a randomized controlled trial, as one of our investigators had earlier established the benefit from counseling by minimally trained counselors in the same community [24]. Another limitation is that a majority of the non-counseled group refused to take the AKUADS after 4 and 8 weeks of initial identification, leaving us with small numbers for comparison. The third limitation is that those women who were enrolled towards the end of the study could not be followed for a longer period of time. This could also possibly be a reason for a lower recurrence rate (16.4 percent). The fourth limitation is that the interviewers were not blind to the counseling status of the women interviewed at 4^th ^and 8^th ^weeks follow-up as they themselves were the counsellors.

In addition, this study was conducted in two underprivileged urban communities; hence, the study participants may not completely represent the city population.

### Recommendations

Depression after childbirth is of great concern to primary and mental health-care professionals. In most developing countries primary care practitioners neither have the time nor the skill to counsel patients; and certified psychologists/counselors are very few. They are mostly restricted to big cities and are inaccessible and unaffordable for most of the population. Moreover, these countries cannot afford the luxury of developing a special cadre of community health workers taking care of mental health problems only. Therefore, it is recommended that minimal skills for identification and counseling for anxiety/depression should be incorporated in the training of community health workers to improve the mental health of women with anxiety and depression in resource-strained countries. Above all, there is widespread stigma and skepticism attached to conventional psychiatric services which act as barriers for use even when available. Hence community-based counseling services, besides being accessible and affordable, would probably be more acceptable.

## Competing interests

The authors declare that they have no competing interests.

## Authors' contributions

NSA conceived and designed the study and prepared the manuscript. BSA designed the study questionnaire and provided intellectual feedback. ISA managed, analyzed, interpreted the data and provided feedback throughout. AKK provided constructive intellectual feedback and participated in the revision of manuscript. All authors read and approved the final manuscript.

## Authors information

Dr. Niloufer S Ali: MBBS, DCH, MCPS, FCPS. Associate Professor, Department of Family Medicine, The Aga Khan University, Karachi, Pakistan.

Dr. Badar S Ali: MBBS, FCPS. Senior Clinical Lecturer, Department of Family Medicine, The Aga Khan University, Karachi, Pakistan.

Mr. Iqbal S Azam: BSc (Honors), MSc (Statistics). Assistant Professor and Coordinator Statistical Consulting Services, Department of Community Health Sciences, The Aga Khan University, Karachi, Pakistan.

Dr. Ali K Khuwaja: MBBS, MCPS, FCPS. Assistant Professor and Convener Research, Department of Family Medicine & Community Health Sciences, The Aga Khan University, Karachi, Pakistan.

## Pre-publication history

The pre-publication history for this paper can be accessed here:

http://www.biomedcentral.com/1471-244X/10/57/prepub
